# Supplementary material for: Accelerando and crescendo in African penguin ecstatic display songs
Source: Ann N Y Acad Sci. 2025 Jun 13;1549(1):112–9. doi: 10.1111/nyas.15383 (PMC12309443; doi:10.1111/nyas.15383)
Supplement: Supplementary file 1 — Supporting Information [file NYAS-1549-112-s001.pdf]

Accelerando and crescendo in African penguin ecstatic display songs – Supplementary material

Taylor A. Hersh[1,2,*], Yannick Jadoul[1,3,4,*], Marco Gamba[5], Andrea Ravignani[1,4,6,7,#], Livio Favaro[5,#]

[1] Comparative Bioacoustics Group, Max Planck Institute for Psycholinguistics, Nijmegen, Netherlands
[2] Marine Mammal Institute, Oregon State University, Newport, Oregon, USA
[3] Artificial Intelligence Lab, Vrije Universiteit Brussel, Brussels, Belgium
[4] Department of Human Neurosciences, Sapienza University of Rome, Rome, Italy
[5] Department of Life Sciences and Systems Biology, University of Turin, Turin, Italy
[6] Center for Music in the Brain, Department of Clinical Medicine, Aarhus University, Aarhus, Denmark
[7] Research Center of Neuroscience "CRiN-Daniel Bovet", Sapienza University of Rome, Rome, Italy

[*] Joint first authors, corresponding authors: taylor.a.hersh@gmail.com, Yannick.Jadoul@uniroma1.it
[#] Joint senior authors

Method S1. Study subjects and song recording protocol

The number of penguins in each colony varied throughout our study (due to deaths, hatches, etc.), but was approximately 23 for Giardino Zoologico di Pistoia, 14 for Zoomarine Roma, and 66 for Zoom Torino. Note that captive African penguins generally have a more flexible breeding cycle than their wild counterparts.[3,4]

At Giardino Zoologico di Pistoia, penguins were housed in an outdoor exhibit measuring 70 m^2, including a saltwater pond of 40 m^2. This colony was established in 2011 via ten adult penguins (9 males, 1 female) transferred from the Warsaw Zoo (Warsaw, Poland). The colony grew in 2012 with the addition of seven birds from the Nausicaá Centre National de la Mer (Boulogne-sur-Mer, France). From 2011–2016, breeding pairs produced six chicks.

At Zoomarine Roma, penguins were housed in an outdoor exhibit measuring 298 m^2, including a saltwater pond of 200 m^2. This colony was established in 2015 by combining adult penguins from Burgers' Zoo (Arnhem, Netherlands) and Y adult penguins from Zoo des Sables d'Olonne (Les Sables-d'Olonne, France) to create a colony of 15 individuals (8 males, 7 females).

At Zoom Torino, penguins were housed in an outdoor exhibit measuring 1,500 m^2, including a freshwater pond of 120 m^2. This colony was established in 2009 by combining 37 adult (approximately evenly numbers of males and females) African penguins that had been hatched in four different zoological facilities: Artis Royal Zoo (Amsterdam, Netherlands), Vogelpark Avifauna (Alphen aan den Rijn, Netherlands), Wilhelma (Stuttgart, Germany), and South Lakes Wild Animal Park (Cumbria, United Kingdom). By 2017, breeding pairs had increased the colony size to 62 individuals.

The diet of African penguins in captivity consists mainly of oily fish, such as anchovies, sardines, and mackerels, which provide the necessary proteins and fats. The fish is often served whole and feeding sessions vary from 2 to 4 per day. In some cases, vitamin supplements, particularly salt and vitamina, are added to ensure a balanced diet and compensate for any deficiencies arising from the fish alone. However, it is impossible to provide a fixed rule for the diet of every colony, as it is at the discretion of local veterinarians and can vary depending on several factors, such as the season, the weight and health of the animals, and the availability of food.

Penguins were recorded using a RØDE NTG-2 super-cardioid microphone (frequency response: 20 Hz–20 kHz, sensitivity: −36±2 dB re 1 V/Pa at 1 kHz, maximum sound pressure level: 131 dB) on a RØDE PG2 pistol grip. The microphone and grip were placed 5–10 m from vocalizing individuals. The microphone output signal was digitized using a TASCAM portable recorder (model: DR-680 or DR-40, sampling rate: 44.1 kHz) and saved to an internal SD memory card in WAV format (resolution: 16 bits).

Method S2: Relative IOI position binning protocol

When calculating how the coefficient of variation (CV) of IOI duration changes throughout songs, the different lengths of the songs complicate grouping the relative positions together. For example, a song with three IOIs will have IOI durations at relative positions 0%, 50%, and 100%, whereas a song with four IOIs will have IOI durations at relative positions 0%, 33%, 66%, and 100%. To be able to calculate how the CV changes throughout songs, we grouped the IOIs into five bins, around 0%, 25%, 50%, 75%, and 100%, and assigned each IOI duration to its respective bin. More precisely, this results in the following bins: [0, 0.125); [0.125, 0.375); [0.375, 0.625); [0.625, 0.875); [0.875, 1].

Audio S1 (separate files). Audio file and annotations of the ecstatic display song featured in Figure 1

This ecstatic display song was produced by penguin P5 from the Giardino Zoologico di Pistoia colony (Table S1). The full audio file (~12.5 s) and corresponding Praat text grid with syllable annotations is provided, but only the portion leading up to and including the first B syllable (~7 s) was included in our analyses.

Table S1. Number of ecstatic display songs recorded from captive male African penguins included in our study

Penguin identity codes from [2] and from the European Association for Zoos and Aquariums (EAZA) are listed for each male.

Colony	*Code from* [2]	*Code from EAZA*	*Number of songs*
Giardino Zoologico di Pistoia	P1	10409	8
	P2	6100	8
	P3	2207	10
	P4	2592	9
	P5	8199	26
	P6	9173	24
	P7	7215	27
	P8	14140	24
Zoomarine Roma	ZMN2	9060	14
	ZMN3	10092	23
	ZMN4	13255	21
	ZMN6	13055	46
	ZMN7	3852	33
	ZMN8	13252	42
	ZMN9	9061	52
Zoom Torino	ZO1	4109	6
	ZO2	13124	8
	ZO3	3930	9
	ZO4	3867	7
	ZO5	7003	7
	ZO6	770	10
	ZO7	4176	13
	ZO8	7006	15
	ZO9	3866	11
	ZO10	773	29
	ZO11	4110	69
**Total**	**26 males**		**551 songs**

Figure S1. Analysis and test of C syllables' contribution to accelerando

When the C syllable onsets are omitted from acceleration ratio calculations, the ratios have a higher likelihood to be drawn from the baseline distribution compared to when the C syllable onsets are included. The median log-likelihood of both distributions, C-omitted and C-included, is significantly different (Wilcoxon signed-rank test, n = 13 penguins, T = 0.0, $p < 0.001$; see main text and Figure 2A), indicating that C syllables do not fulfil the same role as A syllables in a song's temporal structure. Colors denote penguin identity (Figure 2).

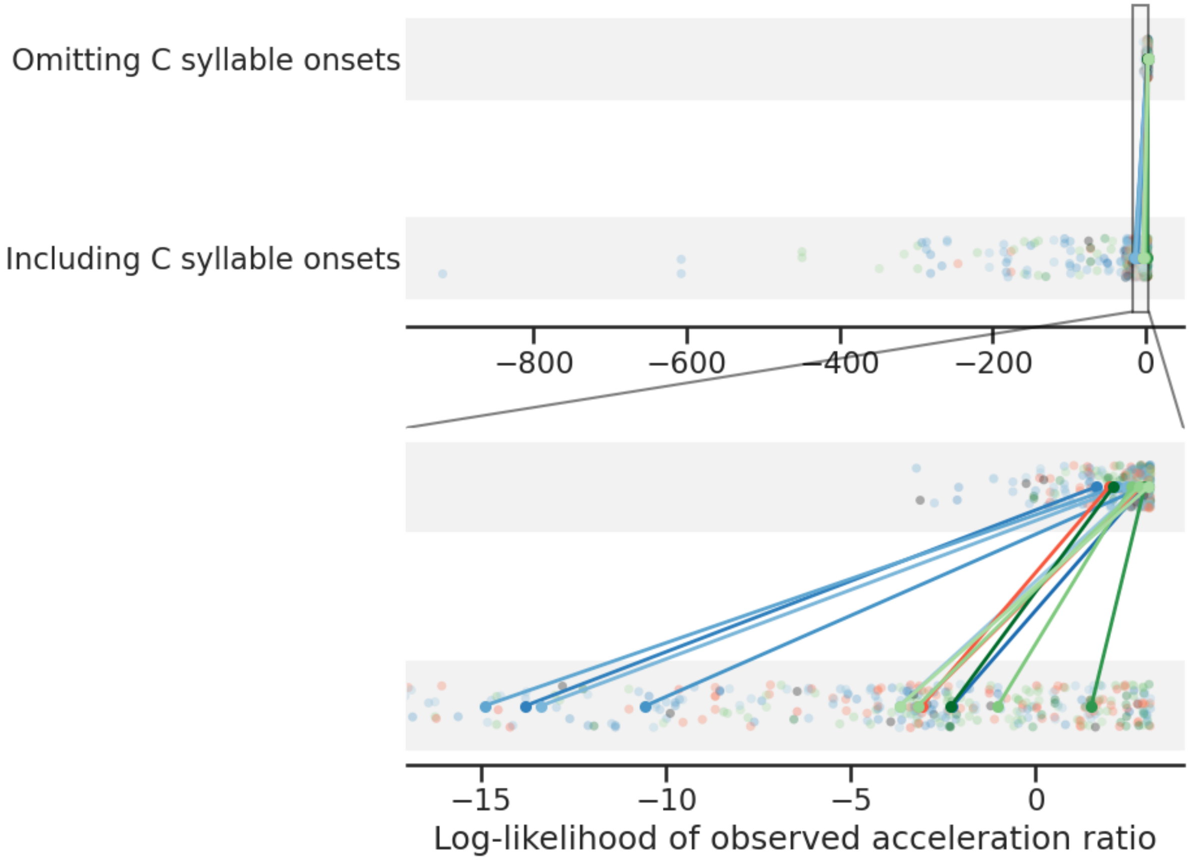

Figure S2. Per-penguin linear regressions for relative IOI position vs. log-IOI

For each of the 26 penguins, the fitted linear regression using all song syllables is highly significant. This confirms the result we find with a single LMEM (see main manuscript): the IOI in a penguin's song is negatively correlated with its relative position within a song. Colors (Figure 2) and alphanumeric codes (Table S1) denote penguin identity.

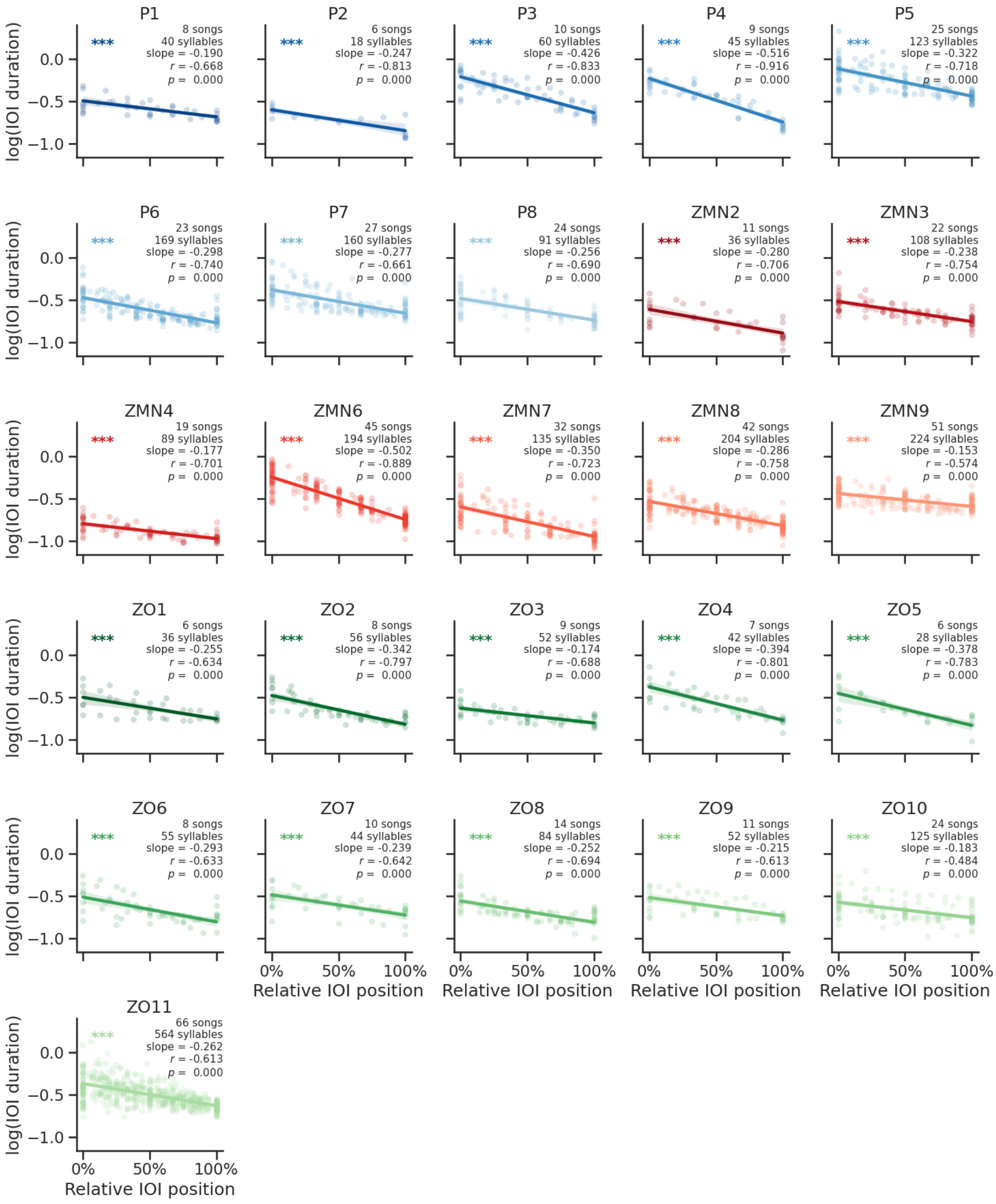

Figure S3. Analysis and test of the change in acceleration across songs

Across penguins, songs accelerate from the start to the end, though acceleration is not completely constant. **(A)** Acceleration is significantly lower at the end of songs than at the start (chi-square test, n = 2253 syllables, $\chi^2(1) = 65.861$, $p < 0.001$), but the effect is small (LMEM estimate: -0.00831 from start to end). Colors denote penguin identity (Figure 2). **(B)** After splitting the acceleration ratios into five bins (see Method S2 for details) based on their relative position within the song, the resulting distribution of acceleration ratios confirms this. In all five bins, the acceleration ratios are significantly higher than 0.5 (i.e., the ratio corresponding to isochrony), showing consistent accelerando (five Wilcoxon rank sum tests; $p < 0.001$ for all). Per each bin, the fraction of acceleration ratios > 0.5, from left to right, is: 83.8%, 79.9%, 82.2%, 81.4%, and 69.6%.

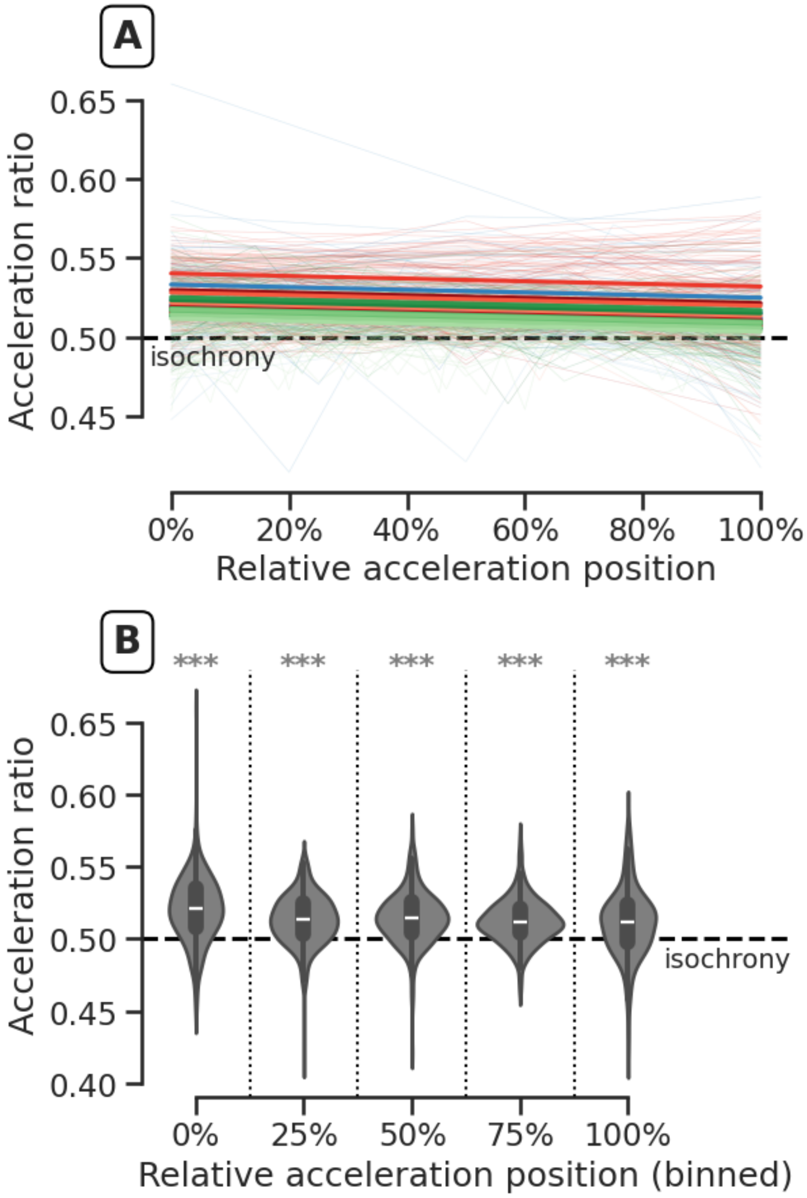

Figure S4. Between- and within-individual variation in song structure and mean acceleration
Several song characteristics vary strongly within penguins, indicating that each penguin does not have a fixed song structure. This is shown by the per-penguin distributions of **(A)** the number of A syllables in a song, **(B)** the number of C syllables in a song, and **(C)** the mean IOI acceleration ratio per song. Not all penguins have an identical range of values, but their distributions overlap considerably and demonstrate that penguins show a certain level of vocal plasticity. Colors (Figure 2) and alphanumeric codes (Table S1) denote penguin identity.

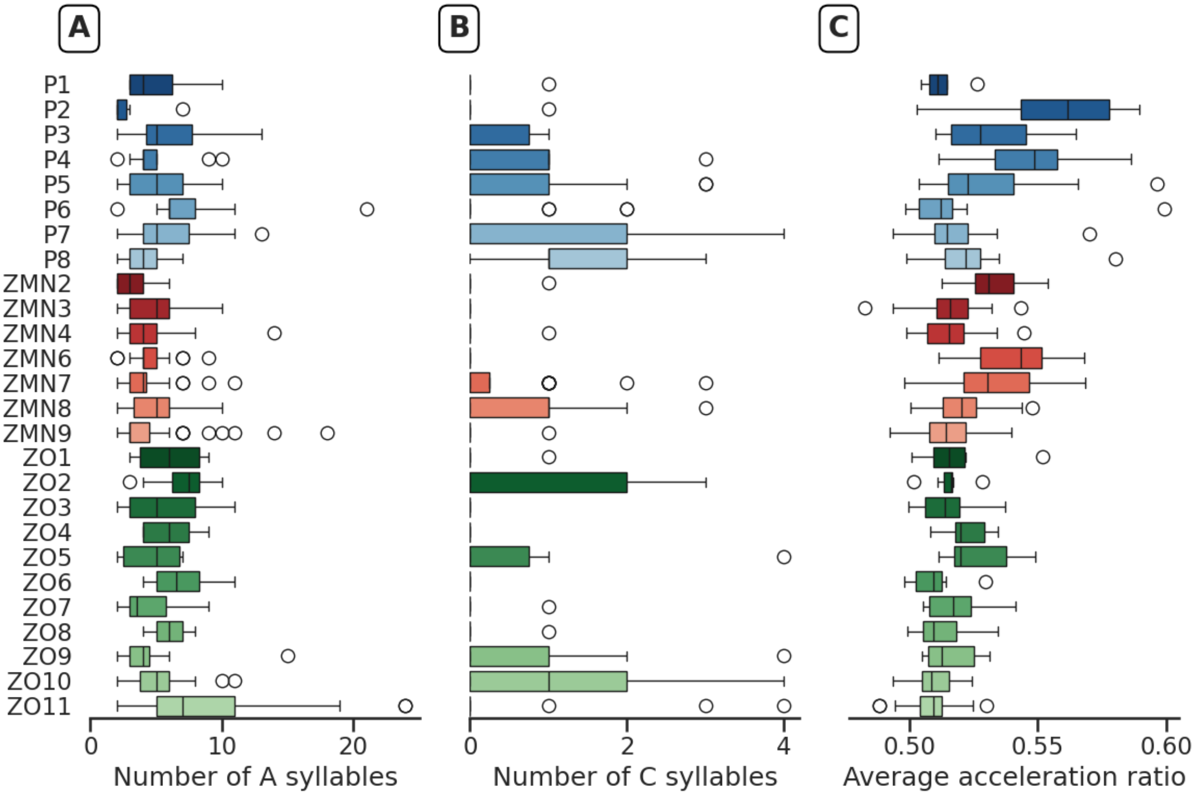

Figure S5. Analysis and tests of the change in duration of IOI components (silences and syllables) as songs progress

LMEMs show the duration of inter-syllable silences decreases throughout songs, whereas the duration of A syllables increases. **(A)** Relative IOI position is negatively correlated with silence duration, with IOIs becoming ~25% shorter and inter-syllable silences decreasing by almost 45% as songs progress (chi-square test, n = 2834 syllables, $\chi^2(1) = 3211.3$, $p < 0.001$). **(B)** In contrast, relative IOI position is positively correlated with A syllable duration: A syllables that occur later in songs are ~20% longer than A syllables that occur earlier in songs (chi-square test, n = 2834 syllables, $\chi^2(1) = 632.55$, $p < 0.001$). Colors denote penguin identity (Figure 2).

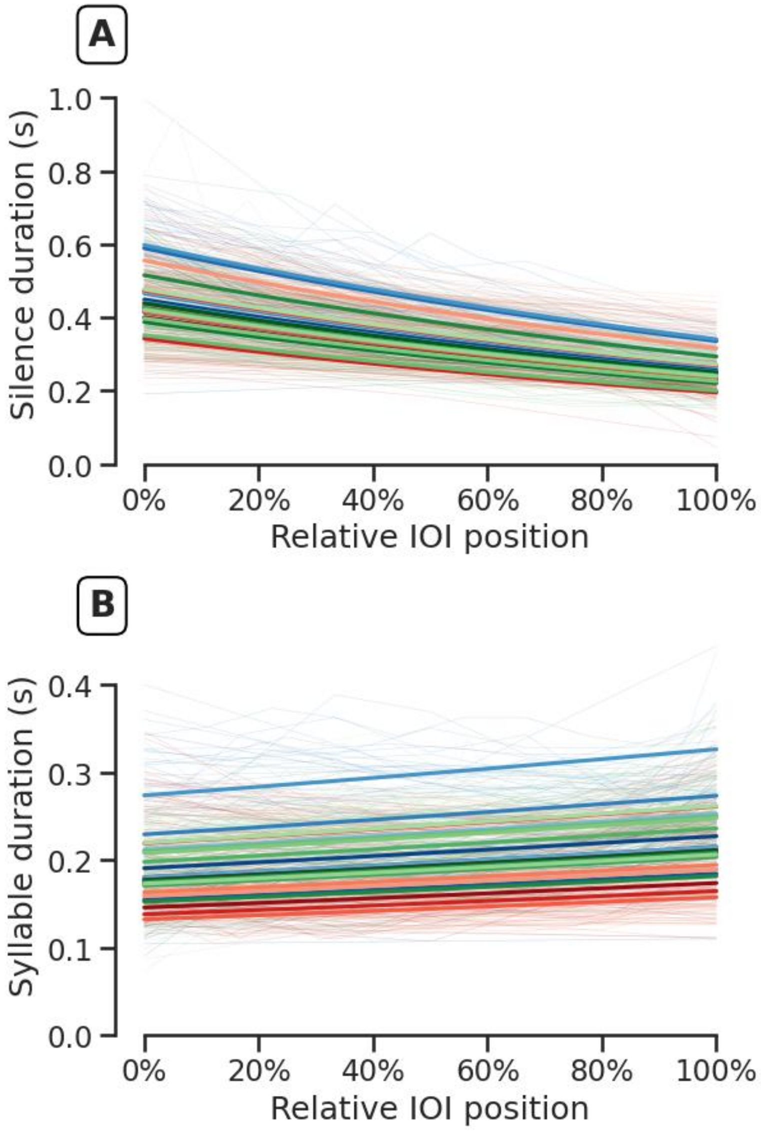

Figure S6. Per-penguin boxplots of the distribution of IOI_1 and IOI_{n-1}

Boxplots of each penguin's IOI_1 and IOI_{n-1} illustrate the reduced variation in IOI duration between the start and the end of a song. Using the modified signed-likelihood ratio test (MSLRT) to compare coefficients of variation (CVs), 13 out of 26 penguins show a significant decrease in CV, even given the limited number of songs for some penguins. Colors (Figure 2) and alphanumeric codes (Table S1) denote penguin identity.

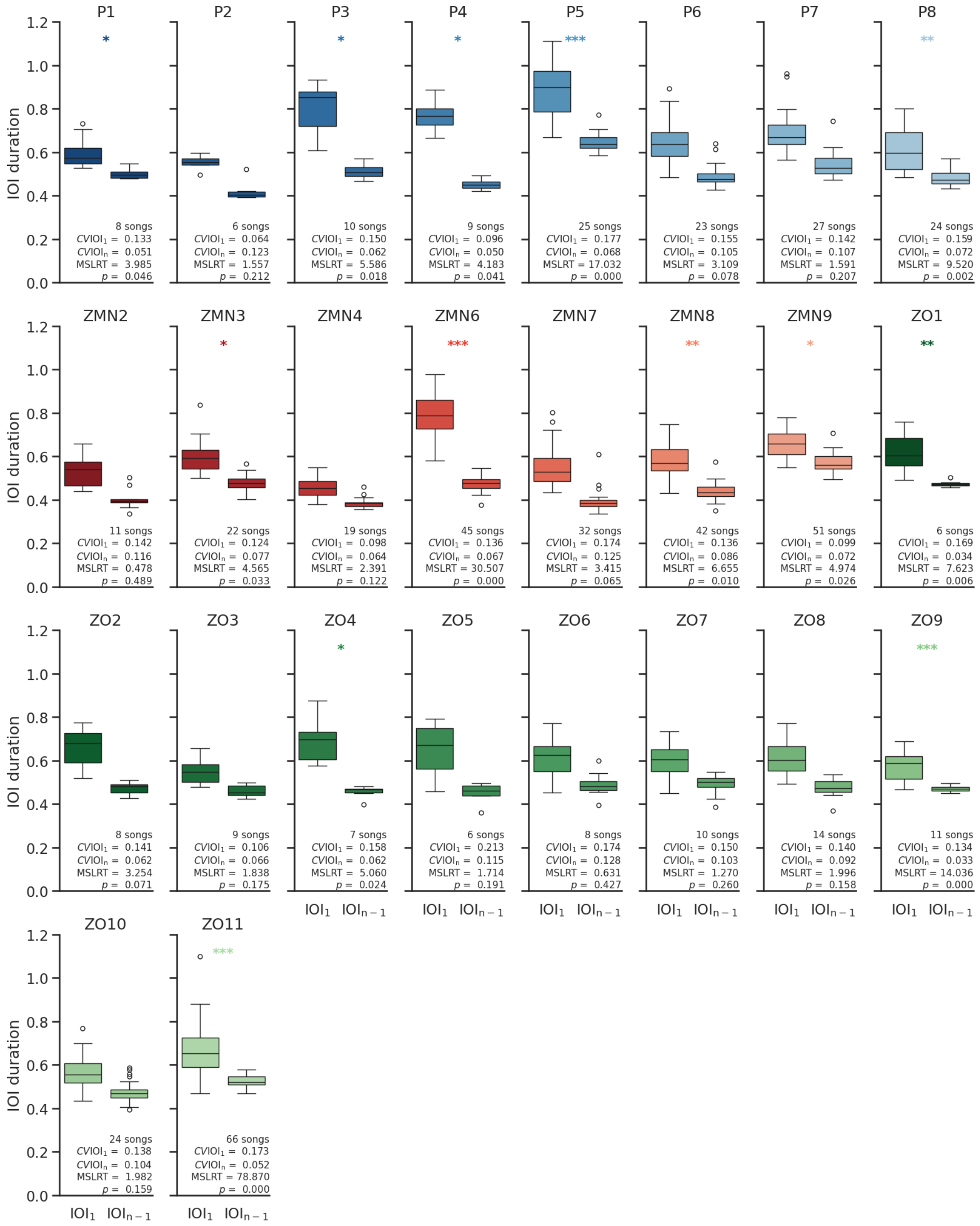

Figure S7. Per-penguin linear regressions for IOI_1 vs. average acceleration after IOI_1

Seven out of 26 fitted linear regressions—one per penguin—show a significant positive correlation between IOI_1 and the average acceleration after IOI_1; 22/26 have a positive slope. This supports the significant result we find when fitting a single LMEM on the combined data (see main manuscript). Colors (Figure 2) and alphanumeric codes (Table S1) denote penguin identity.

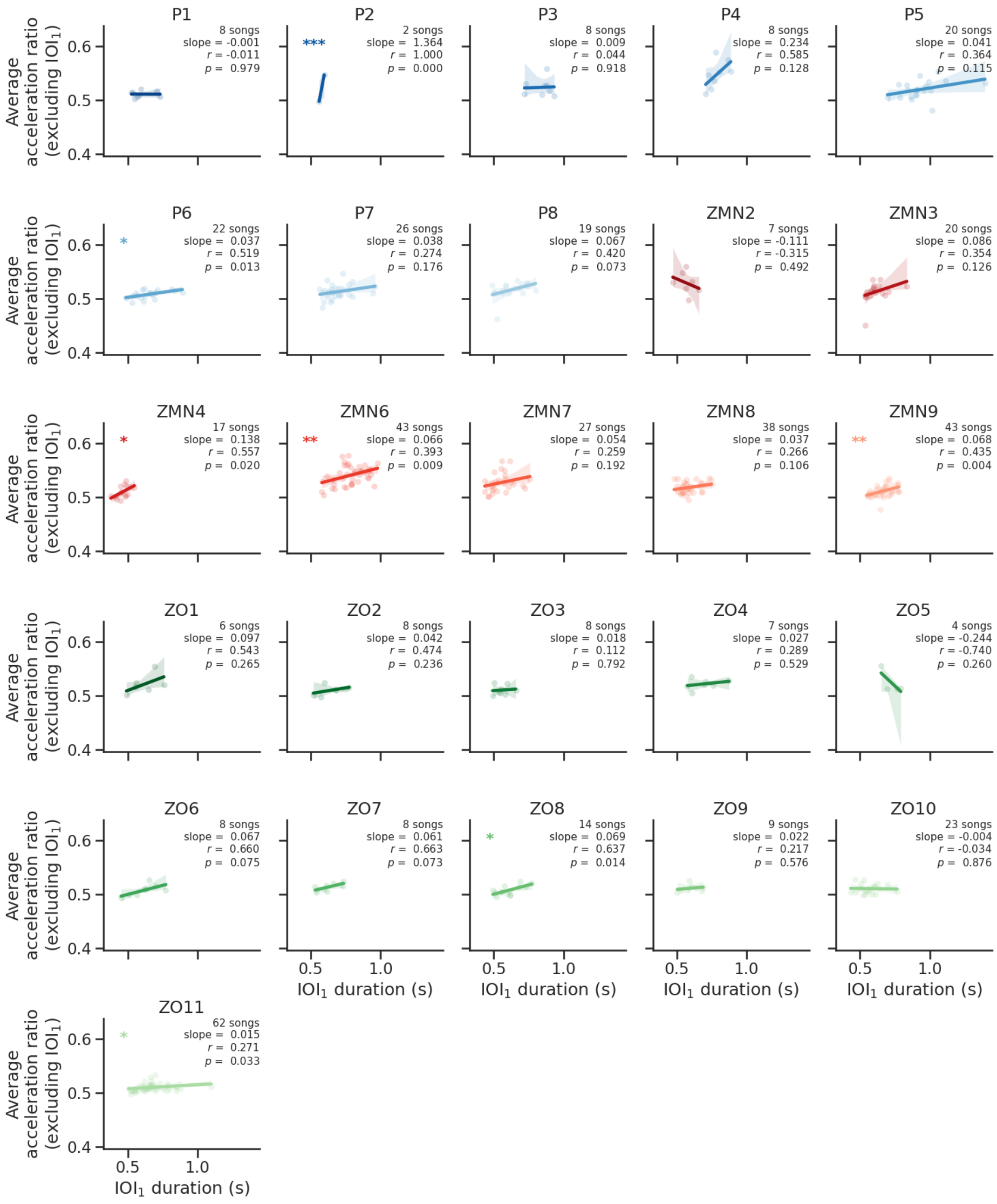

Figure S8. The top five songs of penguin ZMN6 with the longest (top) and shortest (bottom) IOI_1. Songs starting with a long-duration IOI_1 tend to have higher acceleration ratios between adjacent IOIs than songs starting with a short-duration IOI_1. **(A)** The five songs from penguin ZMN6 that have the longest IOI_1 have **(B)** an average acceleration (after IOI_1) between approximately 0.52 and 0.56 (horizontal dashed lines). On the contrary, **(C)** the five songs from the same penguin that have the shortest IOI_1 have **(D)** an average acceleration (after IOI_1) between 0.50 and 0.54. These exemplars demonstrate the significant effect observed in the main text and Figure 2D: songs with a longer IOI_1 generally have a higher average acceleration ratio (excluding IOI_1).

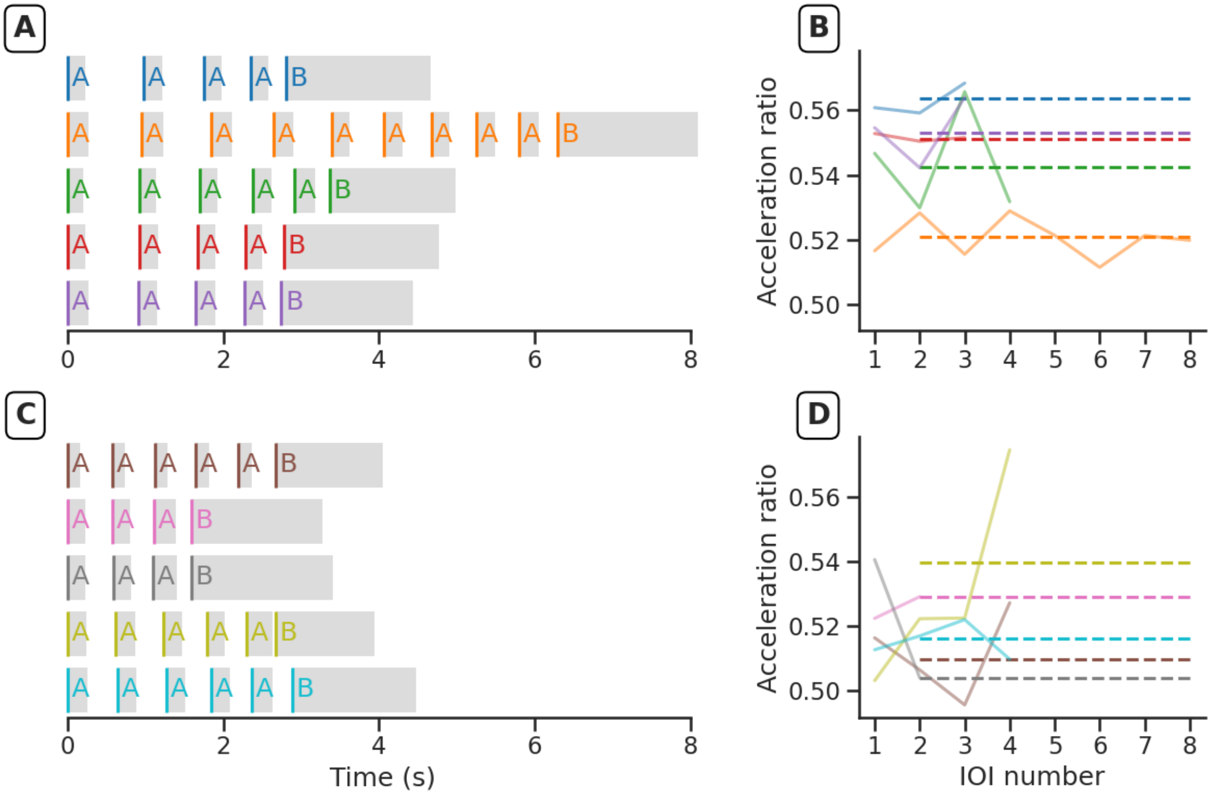

Figure S9. Per-penguin linear regressions for relative syllable position vs. intensity

All fitted linear regressions—one per penguin—show a highly significant correlation between relative syllable position and normalized acoustic intensity. This confirms the significant result we find when fitting a single LMEM on the combined data (see main manuscript). Colors (Figure 2) and alphanumeric codes (Table S1) denote penguin identity.

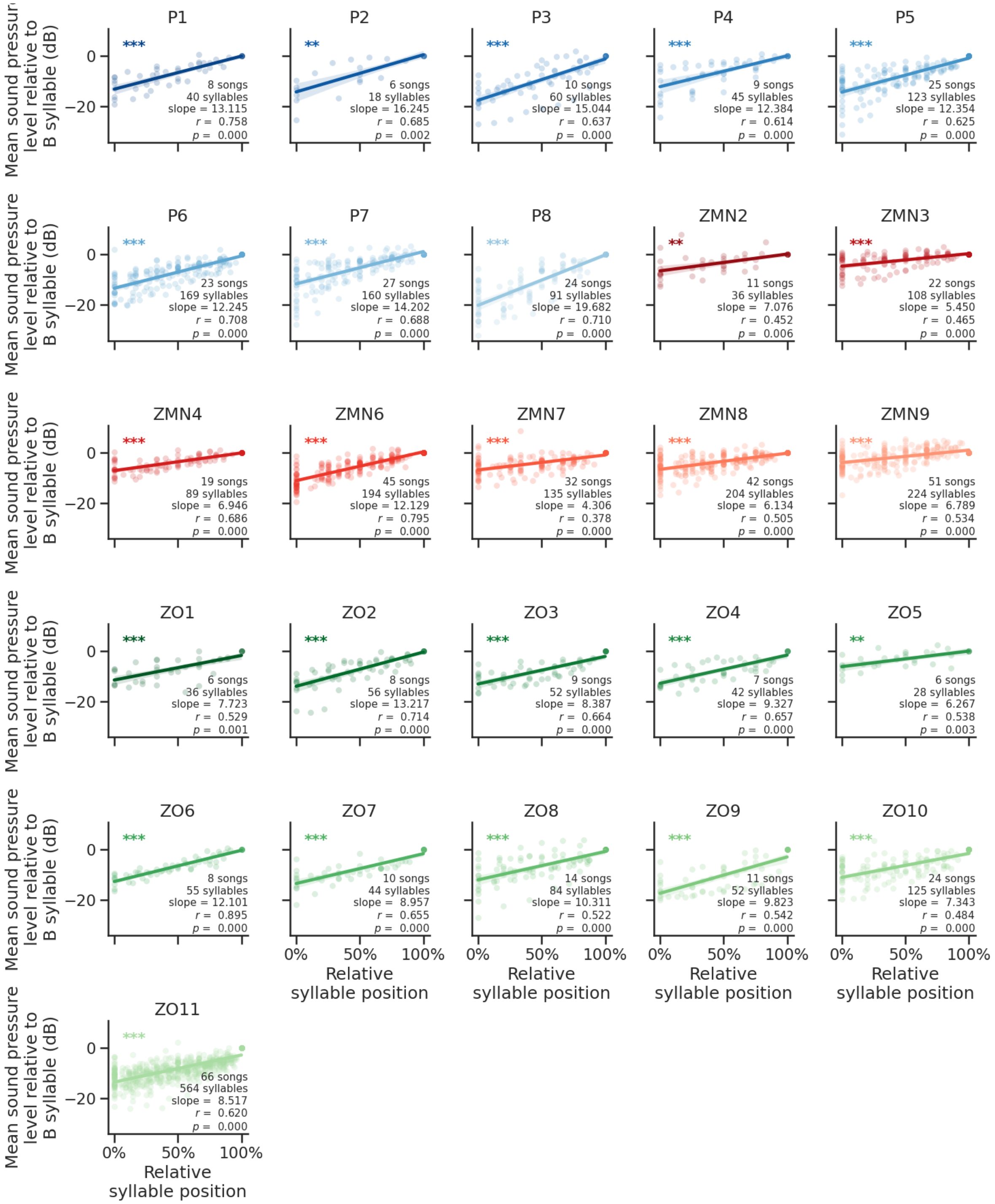

Supplementary references

1. Favaro L., L. Ozella & D. Pessani. 2014. The vocal repertoire of the African penguin (*Spheniscus demersus*): Structure and function of calls. *PLoS One* **9**: e103460. https://doi.org/10.1371/journal.pone.0103460
2. Favaro L., M. Gamba, E. Cresta, *et al.* 2020. Do penguins' vocal sequences conform to linguistic laws? *Biology Letters* **16**: 20190589. https://doi.org/10.1098/rsbl.2019.0589
3. Baciadonna L., C. Pasquaretta, V. Maraner, *et al.* 2024. Network social dynamics of an ex-situ colony of African penguins following the introduction of unknown conspecifics. *Applied Animal Behaviour Science* **273**: 106232. https://doi.org/10.1016/j.applanim.2024.106232
4. Figel T., S.P. Coyne & K. Martin. 2023. Sex and age differences in activity budgets in a population of captive African penguins (*Spheniscus demersus*). *Journal of Applied Animal Welfare Science* **26**: 438–446. https://doi.org/10.1080/10888705.2021.1984916
